# Improving IMES Localization Accuracy by Integrating Dead Reckoning Information

**DOI:** 10.3390/s16020163

**Published:** 2016-01-27

**Authors:** Kenjiro Fujii, Hiroaki Arie, Wei Wang, Yuto Kaneko, Yoshihiro Sakamoto, Alexander Schmitz, Shigeki Sugano

**Affiliations:** 1 Hitachi Industrial Equipment Systems Co., Ltd., 3 Kanda-neribei-cho, Chiyoda-ku, Tokyo 101-0022, Japan; fujii-kenjirou@hitachi-ies.co.jp; 2 Department of Modern Mechanical Engineering, School of Creative Science and Engineering, Waseda University, 3-4-1 Okubo, Shinjuku-ku, Tokyo 169-8555, Japan; arie@aoni.waseda.jp (H.A.); wangwei@aoni.waseda.jp (W.W.); y_kaneko@sugano.mech.waseda.ac.jp (Y.K.); schmitz@aoni.waseda.jp (A.S.); sugano@waseda.jp (S.S.)

**Keywords:** indoor positioning, IMES, pedestrian dead reckoning, hybrid positioning

## Abstract

Indoor positioning remains an open problem, because it is difficult to achieve satisfactory accuracy within an indoor environment using current radio-based localization technology. In this study, we investigate the use of Indoor Messaging System (IMES) radio for high-accuracy indoor positioning. A hybrid positioning method combining IMES radio strength information and pedestrian dead reckoning information is proposed in order to improve IMES localization accuracy. For understanding the carrier noise ratio *versus* distance relation for IMES radio, the signal propagation of IMES radio is modeled and identified. Then, trilateration and extended Kalman filtering methods using the radio propagation model are developed for position estimation. These methods are evaluated through robot localization and pedestrian localization experiments. The experimental results show that the proposed hybrid positioning method achieved average estimation errors of 217 and 1846 mm in robot localization and pedestrian localization, respectively. In addition, in order to examine the reason for the positioning accuracy of pedestrian localization being much lower than that of robot localization, the influence of the human body on the radio propagation is experimentally evaluated. The result suggests that the influence of the human body can be modeled.

## 1. Introduction

Systems providing spatial information have become increasingly important in daily life. The Global Positioning System (GPS), which is considered a standard outdoor positioning method, is widely used in many commercial applications, such as outdoor gear, car navigation systems and automatic vehicle control. However, these devices do not have sufficient accuracy for use in indoor environments, because the radio signals from satellites are attenuated and scattered by walls, roofs and other objects [1,2]. In recent years, the indoor positioning problem has attracted increasing research attention because of the strong demand for indoor location-based services, such as pedestrian tracking, navigation and advertisement [3,4,5].

Many studies have focused on indoor positioning systems using radio signal strength information [6,7,8,9]. Established, commercially available indoor positioning systems include the WiFi fingerprinting method [10,11] and “iBeacon” The WiFi fingerprinting method uses received signal strength (RSS) information from a WiFi radio to identify a receiver location. The advantage of this method is that in many cities, numerous WiFi access points have already been installed, and these can be used as infrastructure for the positioning system. This method provides positioning accuracy of a few meters by finding the best matched pattern between the current received RSS and a pre-built RSS map, which might show a unique pattern in each location. “iBeacon”, which has been developed by Apple Inc., uses Bluetooth low energy (BLE) technology. Apple Inc. has provided an application program interface (API) for iPhone developers to access iBeacon functions, thus enabling them to easily create new services using indoor position information.

The Japan Aerospace Exploration Agency has developed a radio-based indoor positioning system called the “Indoor Messaging System (IMES)” [12,13]. IMES has been developed as a part of the Quasi-Zenith Satellite System (QZSS), and it consists of transmitters that simply broadcast their fixed position as a GPS-compatible signal. Mobile devices that receive the IMES signal can directly decode the position information within the signal reception area (10–20 m in diameter). This positioning method is called “proximity detection” or “range free”. Since the receivers neither calculate the distance from the transmitters nor do the trilateration, like GPS, the positioning is stable, even in indoor environments where multipath interference frequently occurs. Another advantage of IMES is that GPS receivers, the most prevalent devices used for outdoor positioning, can be used with minor firmware modifications because IMES uses a GPS-compatible signal. However, IMES has a disadvantage in that the positioning accuracy is low. Because of the nature of the proximity detection, the positioning accuracy is half of the installation interval of the transmitters; it is usually 5–10 m. This positioning accuracy restricts the applications.

Pedestrian dead reckoning (PDR) is another possible solution to realize indoor positioning [14,15,16,17]. In PDR, the relative displacement in each time interval is calculated using an inertial measurement unit (IMU), such as an accelerometer, gyroscope or even a magnetometer. The absolute displacement can be obtained by accumulating these relative displacements from the location where recording is started. PDR is easy to implement on mobile devices, because the above-mentioned IMUs are common in most smartphones, and no external infrastructure is required; furthermore, calculating the location update is relatively simple. However, a simple PDR solution cannot be used in commercial services, because the measurement error in each time interval is accumulated, and so, the tracking error worsens over time. Furthermore, the initial location should be specified to calculate the absolute displacement, and the localization accuracy depends on this initial information.

In this study, we propose a novel positioning method of IMES with RSS-based trilateration and introduce an integration method of IMES and PDR localization so as to overcome each other’s disadvantages; particularly, we combine the PDR’s relatively good displacement accuracy in a short time with the IMES’s absolute positioning capability by using a Kalman filter. The remainder of this paper is organized as follows. In Section 2, we compare IMES to WiFi, which is also a potential indoor positioning infrastructure. In Section 3, a radio propagation model of IMES is investigated in order to understand the relationship between distance and received IMES radio strength. Based on the obtained radio propagation model, in Section 3, we develop a trilateration algorithm to determine the position using the received IMES radio strength from multiple IMES transmitters. Then, in Section 5, we design a Kalman filter that combines information from the IMES radio and dead reckoning systems (wheel encoders for robots and inertial sensor for humans), and in Section 6, the resulting positioning accuracy is experimentally evaluated. In Section 7, we also investigate the human body influence on the radio propagation. Lastly, in Section 8, we conclude this study and give future work.

## 2. Related Studies

Studies have already investigated hybrid positioning methods in which two or more positioning methods are combined to estimate a user’s location. In particular, WiFi fingerprinting combined with PDR has been well studied. Panyov [18] developed a MEMS-based strapdown inertial navigation system with WiFi signal strength measurement. This system can be implemented in a modern smartphone with built-in MEMS sensors, and it does not require any additional special hardware. Xiao [19] and Frank [20] used a similar approach. These studies used a Kalman filter to integrate the WiFi fingerprinting and IMU data. A particle filter has also been used to integrate information from multiple sources [21]. These WiFi-based methods achieved localization accuracy on the order of meters; however, they require a well-surveyed, pre-built radio strength map to obtain an accurate localization result. This map should be updated regularly because it could change easily upon an access point being newly added or removed and the change of the environment.

In contrast, an IMES radio signal is more suitable for high accuracy indoor positioning, because it carries information of the transmitter location encoded in a GPS-compatible format. Therefore, if the radio propagation properties of IMES are known, a pre-built radio strength map is not required. Furthermore, information of the IMES transmitters’ locations is highly reliable, because these locations are managed by a public organization [22]. The technical significance of this study is to investigate the IMES radio propagation properties, to develop a hybrid positioning method that combines IMES localization and dead reckoning information and to evaluate its positioning performance for mobile robot positioning and pedestrian positioning.

## 3. IMES Radio Propagation Model

A radio propagation model is an empirical mathematical model that is used to describe the characteristics of radio wave propagation with respect to frequency, distance from the source and other conditions. In practice, developing a precise radio propagation model is a difficult and complex process owing to the dynamic and unpredictable nature of the environment. However, the radio propagation model remains the most important model for RSS-based indoor positioning, because it is the most straightforward way to describe the relationship between the RSS and the distance from the transmitter.

To use IMES radio for high accuracy indoor positioning, we need to develop a practical IMES radio propagation model through experiments. In an experiment, an IMES radio transmitter was set at a height of about 2.2 m within an empty indoor environment for broadcasting signals, and an IMES radio receiver was mounted on a mobile robot. The receiver receives the IMES radio signal, as well as its carrier noise ratio (CNR) at different locations within the indoor environment. In this study, the CNR represents the RSS of the IMES radio, and these two expressions are equivalent.

**Figure 1:**
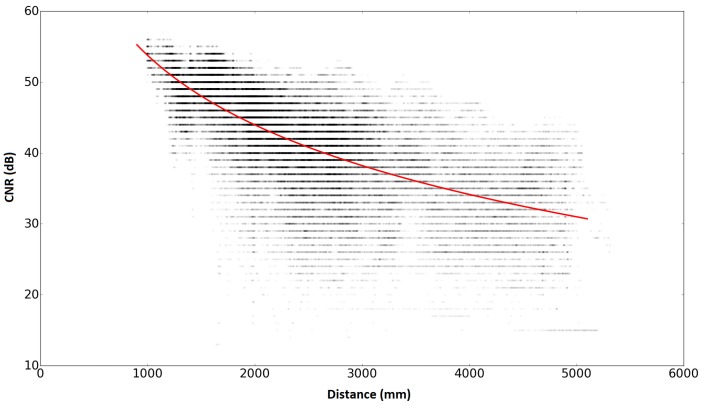
Indoor Messaging System (IMES) radio propagation model.

In our experiment, radio signals were received at over 40,000 points within a 4 m × 4 m area with the IMES transmitter at its center. The distance of each point from the IMES transmitter was calculated. The relationship between the distance and its corresponding CNR measurement is plotted in Figure 1.

The empirical signal path loss model was used to model the relationship between the CNR of the IMES radio and the distance as follows:

(1) $$\begin{array}{cc} cnr & =-10\times \gamma \times log_{10}\sqrt{d^{2}+h^{2}}+\alpha \\ d & =\sqrt{x^{2}+y^{2}} \end{array}$$

where cnr is the received CNR of the IMES radio. As shown in Equation (1), the CNR decreases with the 3D distance between the receiver and the transmitter: $\sqrt{d^{2}+h^{2}}$. The 3D distance is calculated based on the x,y coordinates of the receiver in the horizontal plane and the height *h* of the transmitter. The IMES radio transmitter is set at a height of h=2196 mm in the following experiments; *γ* is the path loss exponent and *α* the constant path loss.

Based on the experimental data plotted in Figure 1, model fitting was performed to obtain the parameters {γ,α} of the path loss model; the results are obtained as γ=32.57,α=151.46. Thus, we obtained the IMES radio propagation model as follows:

(2) $$cnr=151.46-32.57\times log_{10}\sqrt{d^{2}+2196^{2}}$$

This model is used in our indoor positioning method described in the following sections.

## 4. Trilateration Positioning Method

Trilateration is a geometric method that is used to estimate the location of an object by measuring its distances from multiple reference points. It is a well-known technique used in indoor positioning [7,23], and it requires three or more distance measurements to estimate a location in a two-dimensional space.

Based on the IMES radio signal propagation model that was described in Equation (2), the distance from an IMES transmitter can be estimated using a given CNR of the received IMES radio signal. Therefore, the location of an IMES receiver can be estimated by applying trilateration with distance measurements from multiple IMES transmitters.

**Figure 2:**
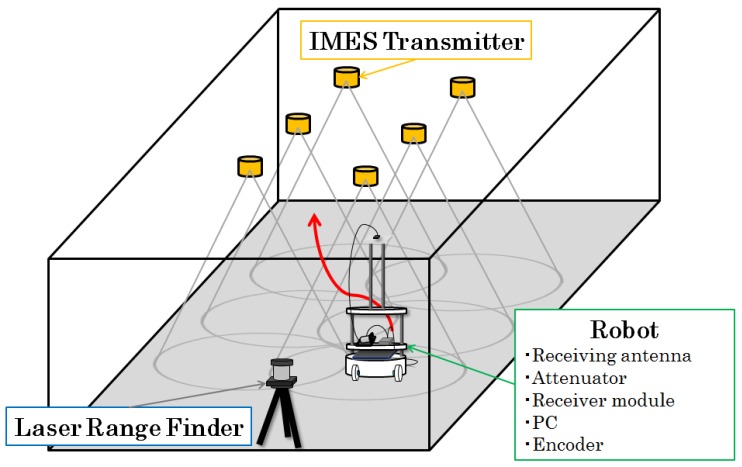
Experimental configuration of IMES-radio-based indoor positioning.

Figure 2 shows the configuration of our test environment for IMES trilateration.

Six IMES radio transmitters are mounted on the ceiling of an indoor environment. These transmitters broadcast the IMES radio modulated with its location and identity information. An IMES receiver is mounted on a mobile robot that moves freely within the indoor environment. The IMES receiver can receive IMES radio signals from multiple transmitters simultaneously and decode the location and identity information of each transmitter. Then, the CNR of the received IMES radio signal is used to estimate its location.

We use the $X_{i},Y_{i}$ coordinate system to denote the location of the *i*-th IMES transmitter, which is known beforehand. x,y denotes the location of a mobile robot or IMES receiver, which is unknown and should be estimated. The CNR of the received signal from the *i*-th IMES transmitter is represented as $cnr_{i}$. *N* denotes the identity number of the IMES transmitter from which the IMES radio signal is received.

Given a set of CNR measurements received, denoted as $\left[cnr_{1},cnr_{2},\cdots ,cnr_{N}\right]$, the corresponding distance $\left[d_{1},d_{2},\cdots ,d_{N}\right]$ can be calculated according to Equation (1) as follows:

(3) $$d_{i}=\sqrt{10^{\frac{\alpha -cnr_{i}}{5\gamma }}-h^{2}}$$

Thus, we can obtain the following observation equations:

d1=(x-X1)2+(y-Y1)2d2=(x-X2)2+(y-Y2)2⋮(4)dN=(x-XN)2+(y-YN)2

**Figure 3:**
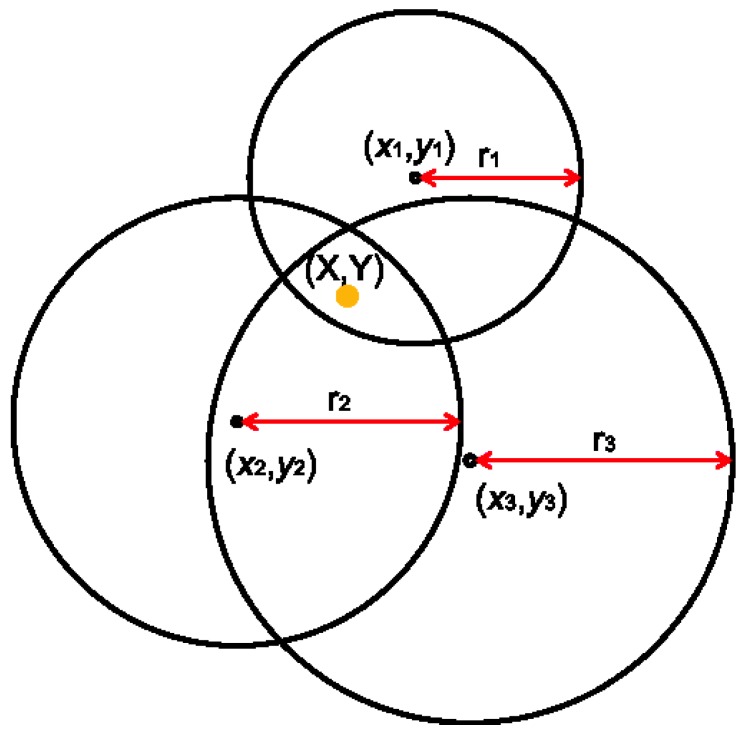
Trilateration problem.

According to Equation (4), the trilateration problem can be formulated as a nonlinear equation solving problem: given the measurement $\left[d_{1},d_{2},\cdots ,d_{N}\right]$ and the known parameters $\left[X_{1},Y_{1},X_{2},Y_{2},\cdots ,X_{N},Y_{N}\right]$, try to find the solution of $\left[x,y\right]$ that best fits the nonlinear Equation (4). Figure 3 shows the trilateration problem.

The empirical Gauss–Newton method can be used to solve the nonlinear equations iteratively. Given an initial estimate of $\left[x,y\right]$ as $\left[x_{0},y_{0}\right]$, the nonlinear equations of Equation (4) can be linearized around $\left[x_{0},y_{0}\right]$, as follows:

d1=d1(x0,y0)+∂d1∂x|(x0,y0)Δx+∂d1∂y|(x0,y0)Δyd2=d2(x0,y0)+∂d2∂x|(x0,y0)Δx+∂d2∂y|(x0,y0)Δy⋮(5)dN=dN(x0,y0)+∂dN∂x|(x0,y0)Δx+∂dN∂y|(x0,y0)Δy

where Δx,Δy is the increase in x,y that will be added to the initial estimate of $x_{0},y_{0}$ to generate the estimate of x,y for the nextiteration step.

According to Equation (5), we can obtain a set of linear equations about Δx,Δy as follows:

(6) $$A\left[\begin{array}{c} \Delta x\\ \Delta y \end{array}\right]=B$$

where A and B are matrices that can be depicted as follows:

(7) $$A=\left(\begin{array}{cc} \frac{x_{0}-X_{1}}{\sqrt{{\left(x_{0}-X_{1}\right)}^{2}+{\left(y_{0}-Y_{1}\right)}^{2}}} & \frac{y_{0}-Y_{1}}{\sqrt{{\left(x_{0}-X_{1}\right)}^{2}+{\left(y_{0}-Y_{1}\right)}^{2}}}\\ \frac{x_{0}-X_{2}}{\sqrt{{\left(x_{0}-X_{2}\right)}^{2}+{\left(y_{0}-Y_{2}\right)}^{2}}} & \frac{y_{0}-Y_{2}}{\sqrt{{\left(x_{0}-X_{2}\right)}^{2}+{\left(y_{0}-Y_{2}\right)}^{2}}}\\ \vdots & \vdots \\ \frac{x_{0}-X_{N}}{\sqrt{{\left(x_{0}-X_{N}\right)}^{2}+{\left(y_{0}-Y_{N}\right)}^{2}}} & \frac{y_{0}-Y_{N}}{\sqrt{{\left(x_{0}-X_{N}\right)}^{2}+{\left(y_{0}-Y_{N}\right)}^{2}}} \end{array}\right)$$

(8) $$B=\left[\begin{array}{c} d_{1}-\sqrt{{\left(x_{0}-X_{1}\right)}^{2}+{\left(y_{0}-Y_{1}\right)}^{2}}\\ d_{2}-\sqrt{{\left(x_{0}-X_{2}\right)}^{2}+{\left(y_{0}-Y_{2}\right)}^{2}}\\ \vdots \\ d_{N}-\sqrt{{\left(x_{0}-X_{N}\right)}^{2}+{\left(y_{0}-Y_{N}\right)}^{2}} \end{array}\right]$$

The linear equations of Equation (6) can be solved to obtain an optimal solution of Δx,Δy using the least squares method, as follows:

(9) $$\left[\begin{array}{c} \Delta x\\ \Delta y \end{array}\right]={\left(A^{T}A\right)}^{-1}A^{T}B$$

The obtained Δx,Δy are then added to the initial estimate $x_{0},y_{0}$ to obtain the estimate x,y within this iteration step, as follows:

x=x0+Δx(10)y=y0+Δy

The obtained x,y is the estimate of the IMES receiver location within this iteration step, and it also serves as the initial estimate of the next iteration step. In our experiment, a satisfactory estimate can typically be obtained after 5–10 iterations.

**Figure 4:**
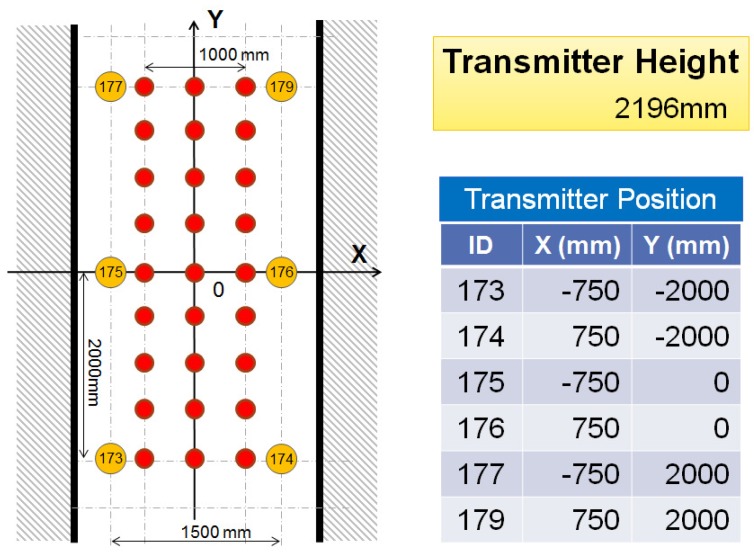
Localization points used for trilateration experiments.

We first conducted an experiment to evaluate the performance of IMES radio-based trilateration without integrating PDR information. During the experiment, 27 points that were uniformly scattered within the environment were adopted as reference locations for IMES radio-based trilateration (see Figure 4).

**Figure 5:**
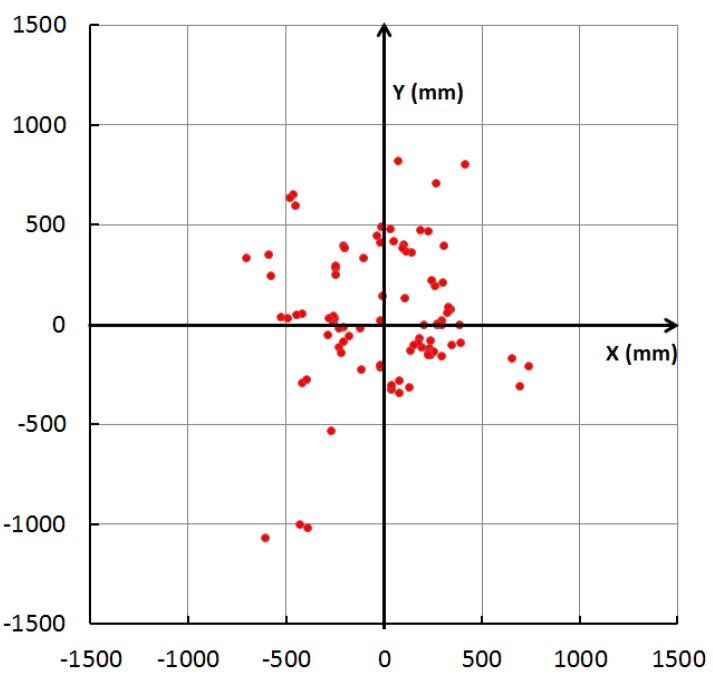
Distribution of the estimation error of IMES radio-based trilateration.

**Figure 6:**
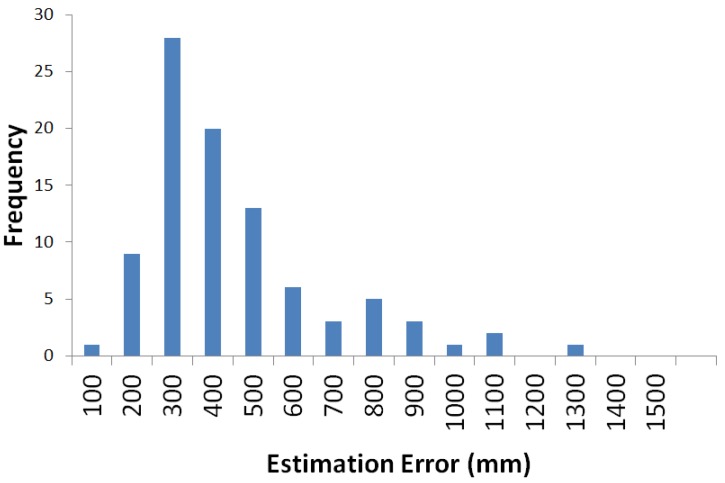
Histogram of estimation error of IMES radio-based trilateration.

In Figure 4, the orange circles represent IMES transmitters. The IMES receiver was fixed at each reference point, as indicated by red circles, to receive IMES radio signals and to use trilateration to estimate its location. Then, the error between the estimated location and the real location was calculated. Figure 5 shows a plot of the distribution of the estimation error. In this figure, each dot represents a single measurement, and it is placed according to the error component in the *x-* and *y*-directions. Figure 6 shows a histogram of the estimation error. Most estimation errors are less than 1 m, and the estimation error was most frequently within 500 mm.

## 5. Integration of IMES Radio and PDR Information

To realize a practical indoor positioning system, dead reckoning technology is introduced because it can provide comparatively accurate measurements of an object’s relative movements if it is in a short period. Other positioning technology, such as radio positioning, will be used to estimate the initial position or to modify the error accumulation during dead reckoning over an extended duration. This combination is meaningful because it is cost efficient and reliable. Thus, we would like to develop an indoor positioning system that combines IMES radio positioning and dead reckoning.

The extended Kalman filter (EKF) is used for integrating IMES radio and dead reckoning, because it is a classic solution for dynamic tracking with different sensor measurements (note that a particle filter also can be used for the multi-sensor integration, but considering the use for embedded systems, we chose the EKF, because its computational load is low). In our system, the IMES radio receiver is mounted on a mobile robot platform that moves randomly within the indoor environment, as shown in Figure 2. The relative displacement of a mobile robot can be measured through its wheel encoder, and the received IMES radio signal is considered a sensor measurement that can be used to modify the dynamic tracking of the robot location. Let x,y denote the coordinate of the mobile robot and *θ* its orientation angle. Thus, the state variable of the system can be written as follows:

(11) $$X={\left[x,y,\theta \right]}^{T}$$

According to the wheel encoders of the mobile robot, we can obtain the relative distance and orientation angle that the robot moved in a time step, denoted as Δd and Δθ, respectively. It can also be considered an input variable U of the system.

(12) $$U={\left[\Delta d,\Delta \theta \right]}^{T}$$

Thus, we could easily obtain the dynamical model of the system as follows:

xk+1=xk+Δdkcos(θk)+ω1yk+1=yk+Δdksin(θk)+ω2(13)θk+1=θk+Δθk+ω3

where superfix *k* and k+1 denote time step *k* and time step k+1, respectively. ***ω*** = ${\left[\omega_{1},\omega_{2},\omega_{3}\right]}^{T}$ denotes the system noise, which is assumed to be drawn from a zero mean multivariate normal distribution with covariance Ω.

The observation equations of the system can be obtained as follows:

d1k=(xk-X1)2+(yk-Y1)2+ψ1d2k=(xk-X2)2+(yk-Y2)2+ψ2⋮(14)dNk=(xk-XN)2+(yk-YN)2+ψN

where $d_{1}^{k},d_{2}^{k},\cdots ,d_{N}^{k}$ is the distance between the robot and the IMES transmitters, which is calculated using the received radio CNR. It is also considered an observation variable Z of the system.

(15) $$Z={\left[d_{1},d_{2},\cdots ,d_{N}\right]}^{T}$$

where *N* is the number of IMES radio transmitters whose radio signal is successfully received. $\left(X_{1},Y_{1}\right),\left(X_{2},Y_{2}\right),\cdots ,\left(X_{N},Y_{N}\right)$ are coordinates of the corresponding IMES radio receivers. $\psi ={\left[\psi_{1},\psi_{2},\cdots ,\psi_{N}\right]}^{T}$ denotes the observation noise, which is assumed to be drawn from a zero mean multivariate normal distribution with covariance **Ψ**.

The dynamical equation and observation equation of the system can also be organized as follows:

Xk+1=f(Xk,Uk,ω)(16)Zk+1=h(Xk,ψ)

This is a typical nonlinear system; thus, we need to use the EKF for state estimation. We could linearize the equations about the current estimated state and then apply Kalman’s equations. The system matrix $F^{k}$ and observation matrix $H^{k}$ can be linearized according to Jacobians as follows:

F=∂f∂X|X=Xk=10-sin(θ)Δd01cos(θ)Δd001H=∂h∂X|X=Xk(17)=x-X1(x-X1)2+(y-Y1)2y-Y1(x-X1)2+(y-Y1)20x-X2(x-X2)2+(y-Y2)2y-Y2(x-X2)2+(y-Y2)20⋮⋮⋮x-XN(x-XN)2+(y-YN)2y-YN(x-XN)2+(y-YN)20

$X^{k}$ denotes the state variable at time step *k*; $X^{k+1|k}$ the estimate of the state variable at time step k+1 given the current state $X^{k}$; $X^{k+1|k+1}$ the final estimate of the state variable at time step k+1. These estimates are assumed to have a Gaussian probability density function, with their covariance matrices defined as $P^{k},P^{k+1|k},P^{k+1|k+1}$, respectively. Let Z and $Z^{k+1}$ denote the observation variable at time step *k* and k+1, respectively; thus, the EKF will generate the estimate $X^{k+1|k+1}$ at time k+1 given its previously estimated $X^{k|k}$ at time *k* and current observation $Z^{k+1}$.

The processing of the EKF can be divided into the prediction process and the update process. During the prediction process, the estimated $X^{k+1|k}$ and its covariance matrix $P^{k+1|k}$ will be predicted according to the following formula:

Xk+1|k=f(Xk,Uk,ω)(18)Pk+1|k=FkPk|kFkT+Ω

During the update process, the final estimate $X^{k+1|k+1}$ and its covariance matrix $P^{k+1|k+1}$ are calculated according to the predicted estimate $X^{k+1|k},P^{k+1|k}$ and the current observation $Z^{k+1}$ according to the following formula:

Xk+1|k+1=Xk+1|k+R(Zk+1-h(Xk+1|k,ψ))(19)Pk+1|k+1=Pk+1|k-RHk+1Pk+1|k

where *R* and *S* are defined as follows:

S=Hk+1Pk+1|kHk+1T+Ψ(20)R=Pk+1|kHk+1TS-1

According to the prediction and update processing of the EKF, the state variables can be tracked continuously in each time step.

## 6. Positioning Experiments

**Figure 7:**
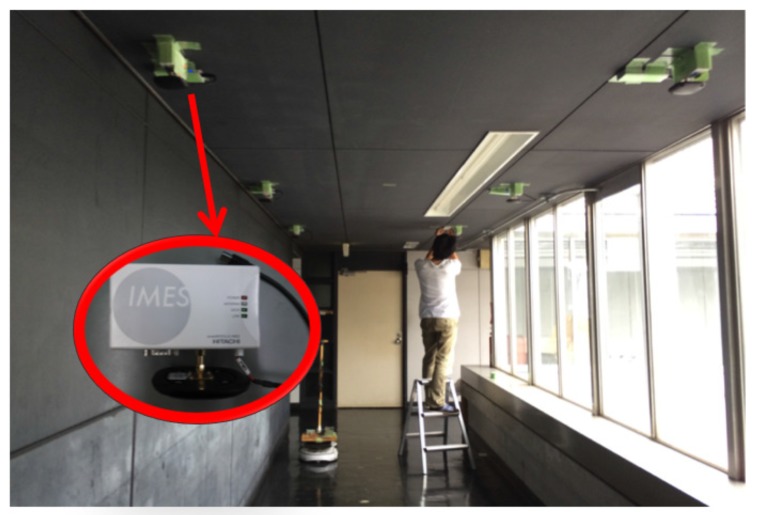
Appearance of the experimental field.

**Figure 8:**
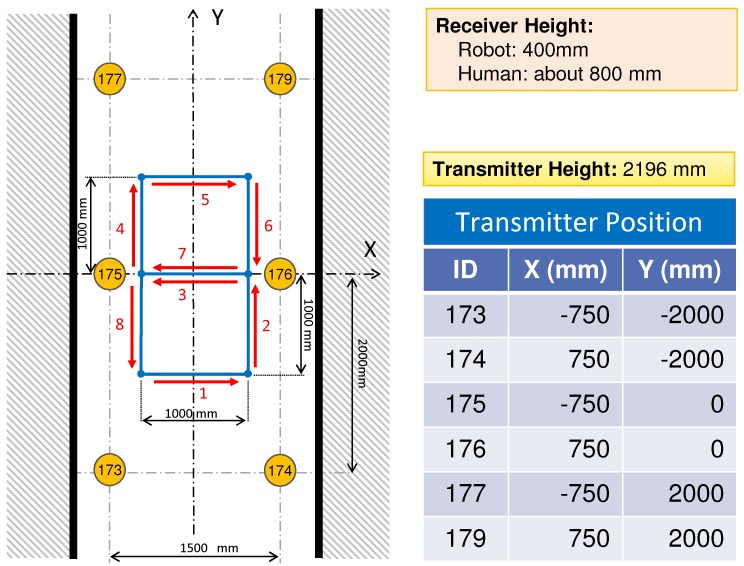
Experimental setup for robot and pedestrian localization.

To evaluate the hybrid positioning method using IMES and PDR information, two positioning experiments, robot localization and pedestrian localization, were conducted. In the following experiments, we used the IMES radio propagation model described in Section 3. Figure 7 shows the appearance of the experimental field. The experiments were performed in the corridor of a university building. Figure 8 shows a schematic of the test environment; it was the same as that in the pre-experiment for investigating the trilateration positioning method described in Section 4. A robot or a human subject followed a figure-eight path indicated by blue lines. As the red arrows and the corresponding numbers represent, it starts moving from the bottom-left point and returns to it. The locations of the transmitters are indicated by orange circles. The numbers in the orange circles indicate the corresponding id numbers of each transmitter. These transmitters were attached on the ceiling as the height of the transmitters was set to 2196 mm. 

The accurate instantaneous location of a robot or a human subject was obtained through a measurement using a laser range finder, and it was considered to measure the actual path. (Note that, in the case of positioning for a human, the path of the receiver antenna was regarded as the human subject’s actual path. To calculate it, the human subject wore a pipe with a diameter of 50 cm, and the center of the pipe was obtained by a positioning algorithm using the laser range finder. Then, in consideration of the offset between the center of the pipe and the receiver antenna on the subject’s hand, the actual path of the receiver antenna was calculated.) The location was estimated via post-processing using three different algorithms: trilateration, dead reckoning and Kalman filtering. The IMES radio signal was received every 100 ms (10 Hz), and the estimation algorithms were calculated in each time interval. The trilateration method used only IMES radio information, and the Kalman filtering method used both IMES radio information and PDR information. In the cases of dead reckoning and Kalman filtering, we provided both correct and incorrect initial position information to the algorithm to evaluate the error correction performance of Kalman filtering.

### 6.1. Robot Localization

#### 6.1.1. Experimental Setup

In this experiment, an IMES radio receiver was mounted on a mobile robot as the height of the receiver was set to 400 mm from the floor. The robot moved according to the pre-defined path shown in Figure 8 at a speed of 100 mm/s and made five round trips. The Kalman filtering method requires a relative distance and an orientation angle to update the position estimation, as described in Section 5. This information can be directly obtained from the wheel encoders installed in the robot used in this experiment at the same sampling rate as the IMES (10 Hz).

The parameters of the EKF were set as follows. For the initial values of the state vector, X, described in Equation (18), (−500 mm, −1000 mm and 0${}^{\circ }$) were set as the correct initial position/orientation, and (−1000 mm, 0 mm and 45${}^{\circ }$) were set as incorrect values. For the initial values of the error covariance matrix, P, in both the cases of the correct and incorrect initial values, (250,000, 250,000, 1) were set to the diagonal elements and zero was set to the off-diagonal elements; this means that the uncertainty of the estimated state is *x* = 500 mm, *y* = 500 mm, and theta = 1 radian as their standard deviation. The system noise covariance matrix, Ω, shown in Equation (18), was set as Ω[0][0] = 0.4, Ω[1][1] = 0.4, Ω[2][2] = 0.000001, and all off-diagonal elements were set to zero. These values were determined empirically based on several dead reckoning results, so that a good EKF estimation result could be obtained. The observation noise covariance matrix, **Ψ**, shown in Equation (20), was set as Ψ[i][j] = 386,001 (i=j) and Ψ[i][j] = 0 (i≠j). Note that the square root of 386,001 is about 621 (the unit is mm); this value is calculated from the data shown in Figure 1 as the weighted average (according to the number of plots) of the standard deviation of the distance for each integer value of the CNR.

#### 6.1.2. Experimental Results

**Figure 9:**
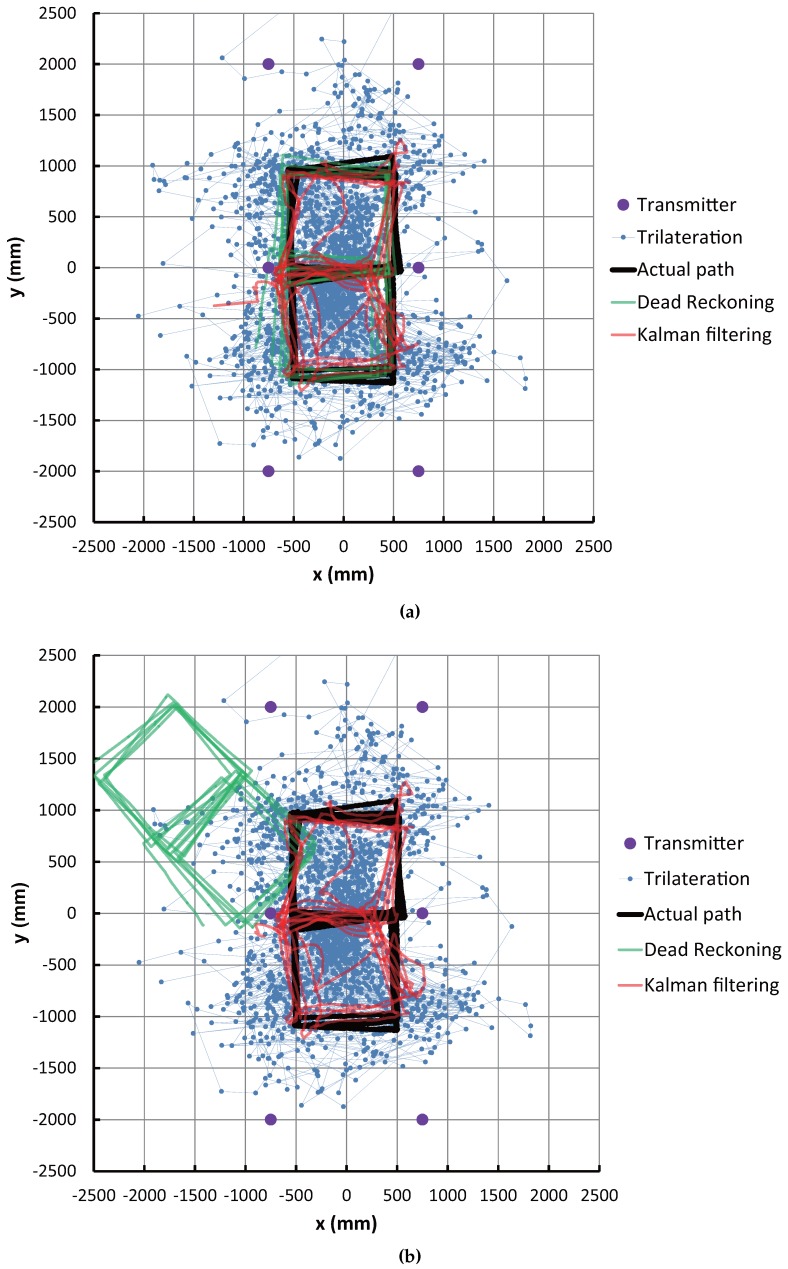
Experimental result of robot position estimation. (**a**) Correct initial position and orientation; (**b**) Incorrect initial position and orientation.

Figure 9a,b shows comparisons between the actual path and the estimated path in the case of using the initial values of correct position and orientation and the case of incorrect values, respectively. In these figures, the purple dot, the blue dot with a line, the black line, the green line and the red line correspond to the transmitter position, position estimated by trilateration, actual path, path estimated by dead reckoning and path estimated by Kalman filtering, respectively.

These results show that the blue dots representing IMES trilateration are widespread (which means low accuracy), but by using the Kalman filtering, the estimation paths are significantly improved. As seen in Figure 9a, the performance of the dead reckoning is higher than the Kalman filtering, even if the convergence period of the EKF in the initial stage of the estimation process is not considered. However, Figure 9b shows that the dead reckoning cannot recover the incorrect initial position and orientation, but the Kalman filtering corrects the wrong initial condition and converges to a nearly correct path.

**Figure 10:**
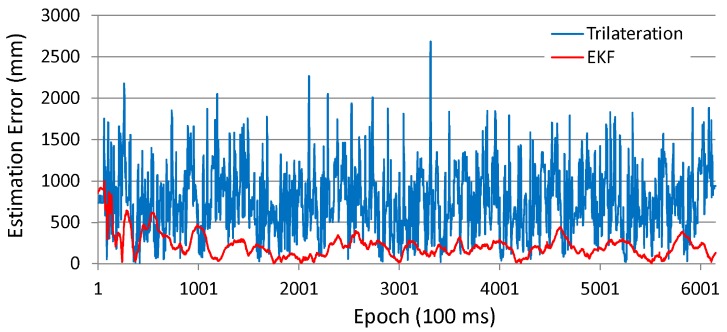
Position estimation error (experiment with robot).

**Figure 11:**
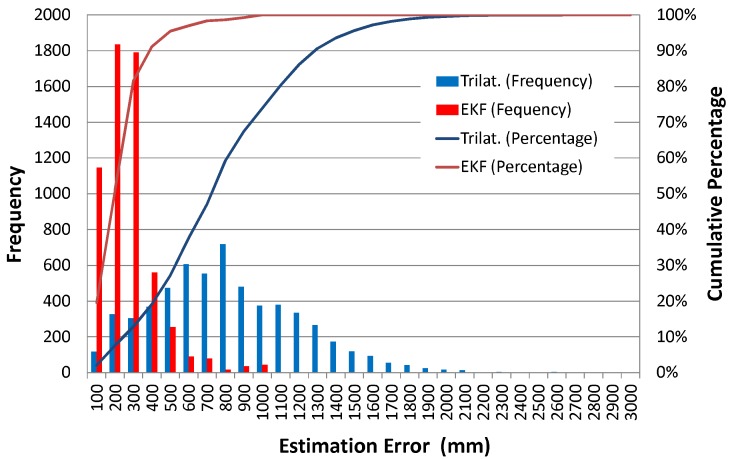
Histogram of position estimation error.

The accuracy of position estimation during path following is also analyzed. Figure 10 and Figure 11 respectively show the time series data of the position estimation errors for both the trilateration and the EKF and their histograms in the case of using incorrect initial values.

As can be seen in these figures, the estimation error of the Kalman filtering case is far smaller than that of the trilateration case. Trilateration has an average estimation error of 766 mm, and an error at the 95 cumulative percentage is 1474 mm, whereas the estimation errors of EKF are respectively 217 mm and 458 mm.

**Figure 12:**
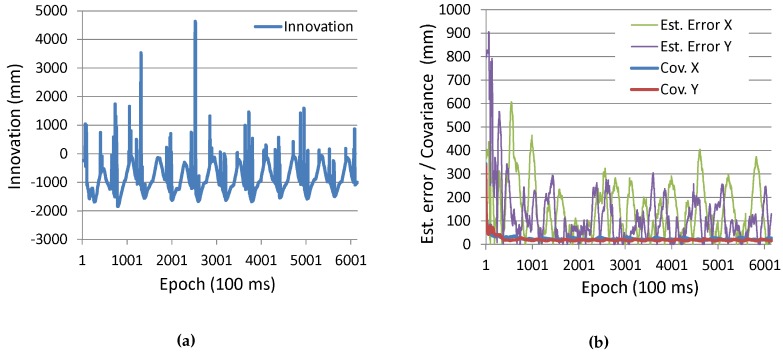
Time series of EKF parameters of robot localization. (**a**) Innovation sequence; (**b**) Estimation error and error covariance.

Figure 12 shows the time series of the parameters of the EKF. Figure 12a shows the sequence of the innovation of transmitter #175 (*i.e*., measurement residual expressed as $Z^{k+1}-h\left(X^{k+1|k},\psi \right)$ in Equation (19), which means the difference between the actual and predicted observation values of the distance between the receiver and transmitter antennas). If the system is properly modeled in the EKF and the system and observation noise are zero-mean Gaussian, the innovation sequence becomes a white noise. However, as seen in the figure, since the sequence has a sort of periodic pattern, such conditions are not completely met. Figure 12b shows the time series of the error covariance, $P^{k+1|k+1}$, represented in Equation (19) and the actual position estimation error. Note that Cov. *X* and Cov. *Y* in the figure actually mean the square root of the diagonal elements of $P^{k+1|k+1}$, which also represent the standard deviation of the uncertainty for the x- and y-components of the estimated position. As seen in the figure, the estimation errors are far larger than the covariance values. This also means that the position estimation algorithm does not completely meet the Kalman filtering conditions mentioned above.

### 6.2. Pedestrian Localization

#### 6.2.1. Experimental Setup

In this experiment, a human subject held an IMES receiver antenna and a motion sensor that includes a three-dimensional accelerometer and a three-dimensional gyroscope (the motion sensor’s sampling rate is 100 Hz). The subject was asked to walk at a speed of 300 mm/s while following the pre-defined figure-eight path shown in Figure 8 fifteen times, and an actual path was measured by the laser range finder as, is the case in the robot localization setup. The reason that the number of round trips was three-times more than the case of robot is because the moving velocity of the human subject was about three-times higher than that of the robot. The height of the IMES receiver antenna was set at approximately-800 mm from the floor, which was assumed to be the height of a subject’s waist.

The PDR information of the relative distance and orientation angle was calculated with the six-dimensional acceleration measurement. Particularly, the relative distance (displacement in an epoch) was calculated with the the number of steps of the subject and his or her stride length. In this case, the sampling rate of the IMES receiver (10 Hz) is higher than the subject’s step rate (1.5–4 Hz); to cope with this, one step was divided into several portions for each corresponding to 100 ms in the post-processing and used for the prediction process of the Kalman filtering. In order to obtain the relative orientation (angular displacement in an epoch), the rotational velocity was acquired from the gyroscope of the motion sensor and it was integrated for 100 ms.

The parameters of the EKF were set as follows. For the initial values of the state vector, X, described in Equation (18), (−500 mm, −1000 mm and 0${}^{\circ }$) were set as the correct initial position/orientation, and (−1000 mm, 0 mm and −45${}^{\circ }$) were set as incorrect values. For the initial values of the error covariance matrix, P, in both the cases of the correct and incorrect initial values, (4,000,000, 4,000,000, 1) were set to the diagonal elements and zero was set to the off-diagonal elements; this means that the uncertainty of the estimated state are *x* = 2000 mm, *y* = 2000 mm, and theta = 1 radian as their standard deviation. The values of the x- and y-coordinates are larger than the case of the robot experiment above. This is because the position estimation error of pedestrians is larger than that of the robot. The system noise covariance matrix, Ω, shown in Equation (18), were set as Ω[0][0] = 2.28, Ω[1][1] = 2.28, Ω[2][2] = 0.000001, and all off-diagonal elements were set to 0. As is the case of robot, these values were determined empirically based on several dead reckoning results so that a good EKF estimation result could be obtained. The observation noise covariance matrix, **Ψ**, shown in Equation (20), were set as Ψ[i][j] = 386,001 (i=j) and Ψ[i][j] = 0 (i≠j). This is the same as the case of the robot experiment.

#### 6.2.2. Experimental Results

**Figure 13:**
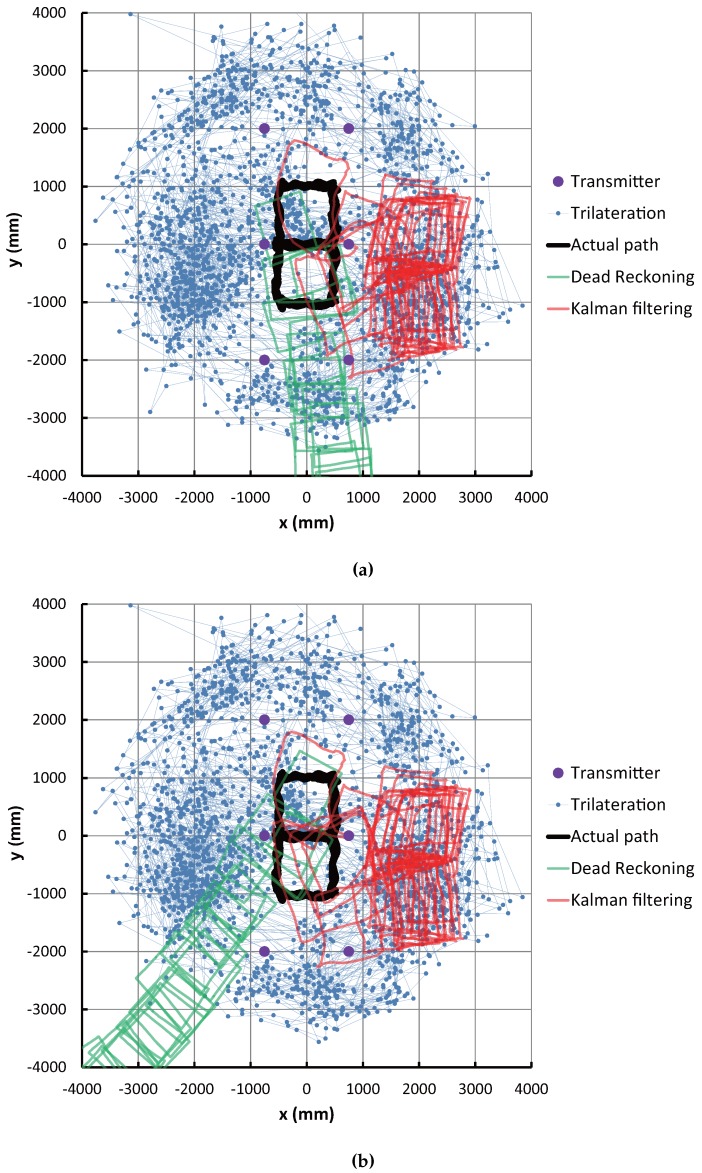
Experimental result of pedestrian position estimation. (**a**) Correct initial position and orientation; (**b**) Incorrect initial position and orientation.

Figure 13a,b shows the results of pedestrian localization in the case of using the initial values of correct position and orientation and the case of incorrect values, respectively. As can be seen in the figures, the performance of the dead reckoning is lower than the case of the robot experiment; because of this, the path drifts quickly toward the bottom of the figures. In contrast, the path of the Kalman filtering remains near the experimental area by utilizing the IMES radio information. However, in both cases of Kalman filtering and trilateration, the positioning accuracy was far lower than that of the robot experiment.

**Figure 14:**
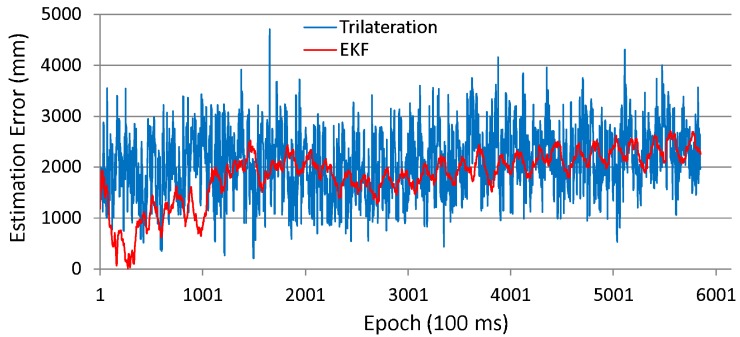
Position estimation error (experiment with human).

**Figure 15:**
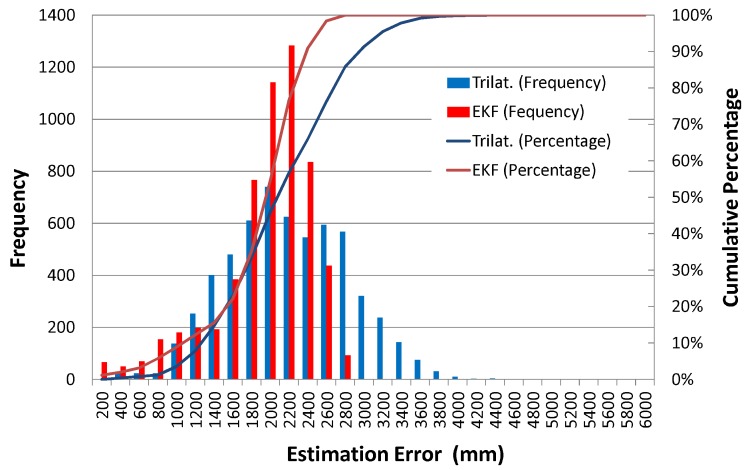
Histogram of pedestrian position estimation error.

Figure 14 and Figure 15 respectively show the time series data of the position estimation errors for both the trilateration and the EKF and their histograms in the case of using incorrect initial values. As can be seen in Figure 14, the estimation error of the EKF follows the trend of the trilateration, although the EKF’s variance is smaller. Figure 15 also shows that there is not a large difference between the average estimation error of the EKF and that of trilateration, compared to the case of the robot experiment. The average estimation error and the 95 cumulative percentage error were respectively 2102 mm and 3179 mm in the case of trilateration and 1846 mm and 2480 mm in the Kalman filtering case.

**Figure 16:**
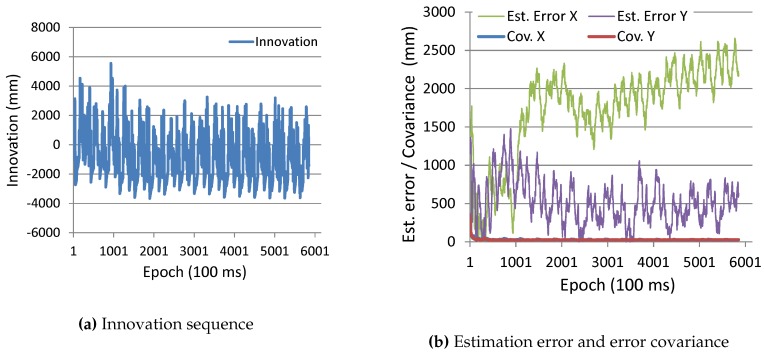
Time series of EKF parameters of pedestrian localization.

As is the case of the experiment with the robot, Figure 16a shows the sequence of innovation of transmitter #175, and Figure 16b shows the time series of the error covariance and the actual position estimation error. Similar to the experimental results with the robot, the innovation sequence is not a white noise, and the estimation errors are far larger than the covariance values (rather, the estimation error is diverging).

### 6.3. Discussion

In the experiment with the robot, a good positioning result was obtained by combining the dead reckoning (which is accurate in a short time) with the IMES radio-based positioning (which is not accurate, but gives an absolute position) with the EKF. However, in consideration of the results shown in Figure 12, the Kalman filtering might not be completely suitable for the IMES radio-based positioning with CNR. The Kalman filtering method basically assumes a zero-mean Gaussian noise for both the prediction and correction process. However, the error of the robot’s wheel encoders (slippage) would not be a zero-mean Gaussian, and as seen in Figure 1, the horizontal distribution of distance plots centering the red line is also not a Gaussian distribution. Therefore, in order to increase the positioning accuracy, it might be better to use an estimator that can cope with a non-Gaussian noise, such as a particle filter.

In the pedestrian experiment, the EKF could reduce the error accumulation caused by the dead reckoning, but the estimated position was biased to the right side, as shown in Figure 13, and caused a large positioning error. As also can be seen in Figure 13, the trilateration performance was very low; especially, the position estimation results were distributed outside the actual path. One possible reason for the low trilateration accuracy is that, because of the existence of a human body between the transmitter and receiver antennas, the power of the IMES radio (CNR) decreased, and as a result, the distance between transmitter and receiver antennas was calculated as longer than the actual distance. In this case, probably, it is difficult to model the noise distribution, because it varies according to the pedestrian posture; that is, to cope with this problem, a more complicated system model would be required.

## 7. Experiment of the Interference of the Human Body

The experimental results of the previous section suggest that the human body interferes with the IMES radio propagation. To clarify its influence, the variation of the CNR according to the positional relation between the transmitter and receiver antennas and the human body is investigated in this section.

### 7.1. Experiment

**Figure 17:**
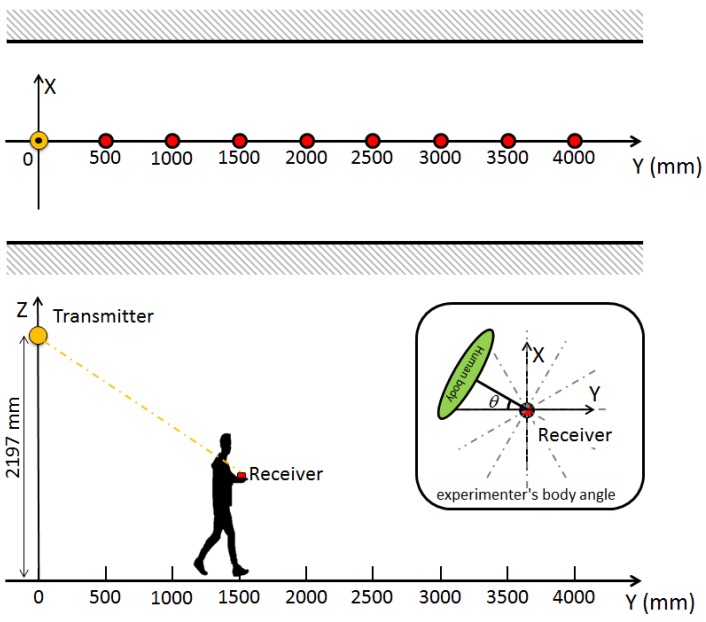
Experimental setup for human body interference.

The overview of the experiment is shown in Figure 17. The experimental procedure is as follows. In the same place used in the experiment shown in the previous section, an IMES transmitter is installed on the ceiling. The experimenter who holds the receiver antenna at the height of 800 mm from the ground moves from the position just below the transmitter antenna (origin of the coordinate system shown in Figure 17) to the position of *y* = 4000 mm. At the points of every 500 mm on the *y*-axis, the experimenter rotates around the receiver antenna with the step size of 30${}^{\circ }$ and acquires the CNR for each angle for 50 epochs while standing still. The positional relation where the experimenter is between the transmitter and receiver antennas corresponds to 0${}^{\circ }$ and 360${}^{\circ }$, and the positional relation where the transmitter antenna, the receiver antenna and the experimenter are on the same line in this order corresponds to 180${}^{\circ }$.

**Figure 18:**
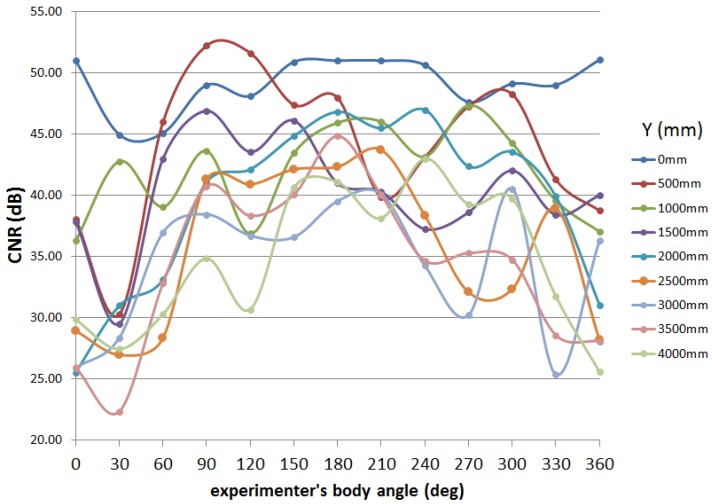
Experimental result of human body interference.

The relation between the CNR and the experimenter’s body angle for each measurement position is shown in Figure 18. As seen in the graph, in the case that the experimenter does not come between the transmitter and receiver antennas (around 180${}^{\circ }$), the CNR is high; on the other hand, in the case of the interference of the experimenter’s body (zero or 360${}^{\circ }$), the CNR decreases. The range of CNR variation according to the experimenter’s body angle is about 5–15 dB. As also can be seen in the graph, the CNR does not greatly vary around the position below the transmitter antenna, but it widely varies in the place far from the transmitter.

### 7.2. Discussion

In consideration of the experimental results mentioned above, it is clear that the human body influences the CNR. Unless its influence can be mitigated, IMES trilateration cannot perform well. One possible solution is to model the influence of the human body and use it to correct the acquired CNR. However, there is a chicken-and-egg problem here; that is, the correct position and orientation of a pedestrian need to be known to correct the body-influenced CNR, but the corrected CNR is necessary to estimate proper position and orientation. In order to overcome this problem, an accurate human body model and a well-performed PDR system would be necessary.

## 8. Conclusions

In this study, we introduced a hybrid positioning method that integrates IMES radio strength information and PDR information to deal with the problems of indoor positioning. The signal propagation of the IMES radio was modeled and identified in order to understand its CNR *versus* distance relation. The result of the pre-experiment showed that IMES radio-based trilateration using the radio propagation model worked well in the test indoor environment with sub-meter-level accuracy. With this result, we tried to improve the localization accuracy by using a hybrid positioning method. The trilateration and the hybrid positioning method were evaluated by applying them to robot localization and pedestrian localization in a test environment. Experiments showed that the hybrid positioning method can gradually rectify the estimation error arising from an incorrect initial position, and it achieved an average estimation error of 217 mm for robot localization. However, the proposed method was not effective for pedestrian localization because of the influence of the human body on the radio propagation. The result of the experiment conducted to investigate the human body influence suggests that it can be modeled. The improvement of pedestrian localization based on such a model is future work.
